# First photographic records of the giant manta ray *Manta birostris* off eastern Australia

**DOI:** 10.7717/peerj.742

**Published:** 2015-01-22

**Authors:** Lydie I.E. Couturier, Fabrice R.A. Jaine, Tom Kashiwagi

**Affiliations:** 1 School of Biomedical Sciences, The University of Queensland, St Lucia, Australia; 2 Climate Adaptation Flagship, CSIRO Marine and Atmospheric Research, Dutton Park, Australia; 3 Biophysical Oceanography Group, School of Geography, Planning and Environmental Management, The University of Queensland, St Lucia, Australia; 4 Marine Megafauna Foundation, Manta Ray & Whale Shark Research Centre, Tofo Beach, Inhambane, Mozambique; 5 Molecular Fisheries Laboratory, The University of Queensland, St Lucia, Australia

**Keywords:** Mobulidae, Manta ray, Australia, Citizen science

## Abstract

We present the first photographic evidence of the presence of the giant manta ray *Manta birostris* in east Australian waters. Two individuals were photographed off Montague Island in New South Wales and off the north east coast of Tasmania, during summer 2012 and 2014, respectively. These sightings confirm previous unverified reports on the species occurrence and extend the known distribution range of *M. birostris* to 40°S. We discuss these findings in the context of the species’ migratory behaviour, the regional oceanography along the south east Australian coastline and local productivity events.

## Introduction

Manta rays (*Manta* spp.) are amongst the largest filter-feeding elasmobranch fishes and have a circumglobal distribution through tropical and temperate coastal waters, offshore islands and seamounts (Marshall, Compagno & Bennett, 2009). Manta rays belong to the family Mobulidae, comprising the two genera *Manta* Bancroft, 1829 and *Mobula* Rafinesque, 1810. All mobulid species are epipelagic zooplanktivores that are presumed to be long lived (e.g., >30 years for *Manta* spp.) and have low fecundities (i.e., late maturity, long gestation period and only a single large pup) (Couturier et al., 2012). Previously considered to be monospecific (*Manta birostris*), the genus *Manta* was redescribed in 2009 to comprise two distinct species: the reef manta ray *Manta alfredi* (Krefft, 1868) and the giant manta ray *Manta birostris* (Walbaum, 1792), and a third putative species *M*. cf. *birostris* (Marshall, Compagno & Bennett, 2009). Both recognised species have circumglobal distributions, sympatric in some areas and allopatric in others (Kashiwagi et al., 2011). *Manta birostris* is considered a more oceanic and migratory species, and is found predominantly in cooler, temperate to subtropical waters (Marshall et al., 2011). *Manta alfredi* displays a high degree of site fidelity in tropical and subtropical waters, but may also undertake local to regional-scale (>700 km) movements and seasonal migrations (Dewar et al., 2008; Couturier et al., 2011; Deakos, Baker & Bejder, 2011; Marshall, Dudgeon & Bennett, 2011; Couturier et al., 2014; Jaine et al., 2014).

Both manta ray species and four of the nine described *Mobula* species are reported to occur in tropical to temperate waters of Australia (Last & Stevens, 2009; Marshall, Compagno & Bennett, 2009). While the occurrence of *M. alfredi* has been widely confirmed off the coast of eastern Australia (Couturier et al., 2011; Couturier et al., 2014), the occurrence of *M. birostris* in this region has been lacking photographic validation despite records in literature (Hutchins & Swainston, 1986; Allen et al., 2006; Last & Stevens, 2009). The recent separation in the genus *Manta* spp. means that records of *M. birostris* prior to 2009 lacking photographic evidence cannot be validated, as species may have been confused with *M. alfredi*. This paper presents the first photographic evidence confirming the occurrence of *M. birostris* in east Australian waters, with one specimen photographed off Montague Island, New South Wales, in January 2012 and one specimen photographed off the northeast coast of Tasmania in January 2014.

## Materials and Methods

**Figure 1:**
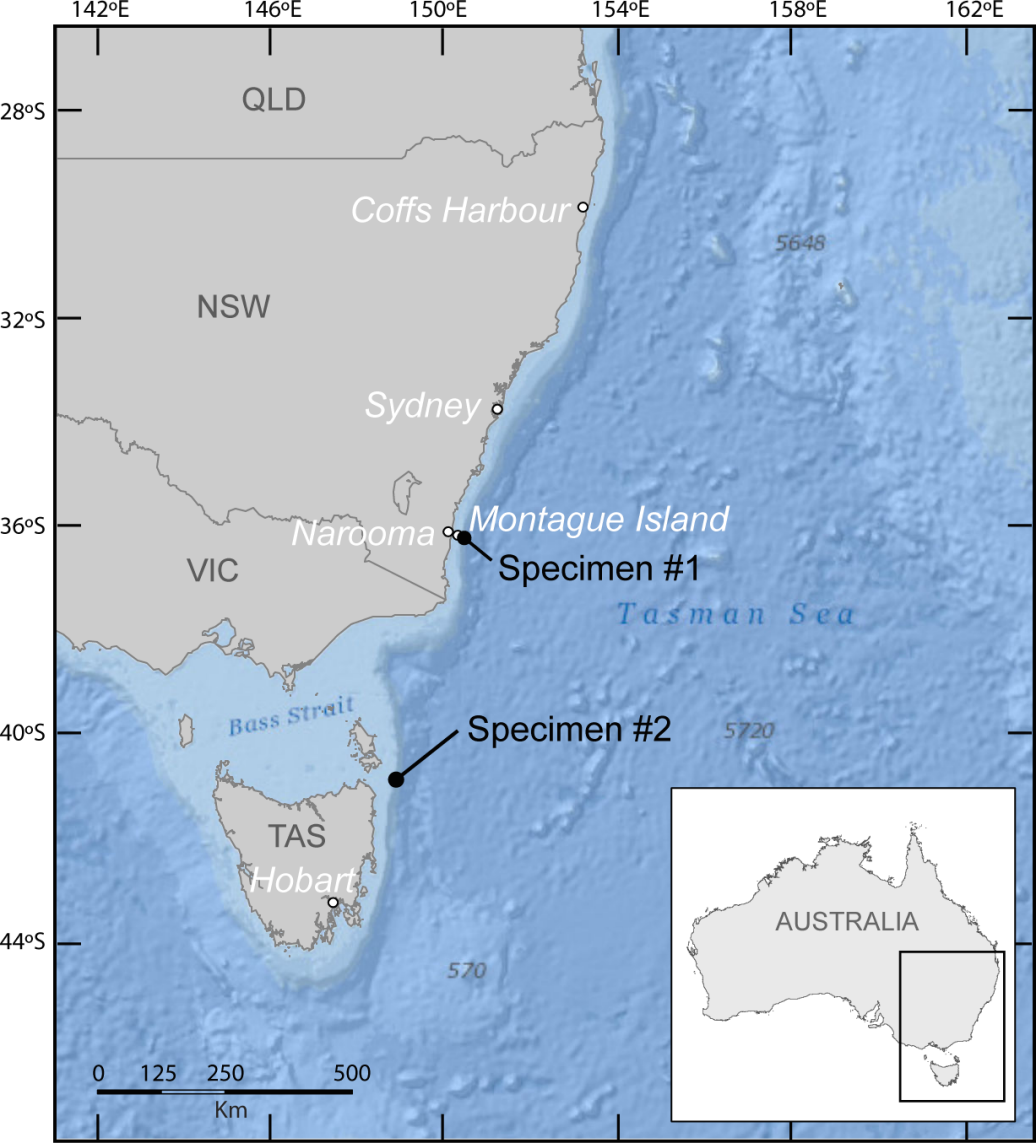
Map of south east Australia showing sighting locations of specimen #1 (Montague Island) and specimen #2 (north east Tasmania).

**Figure 2:**
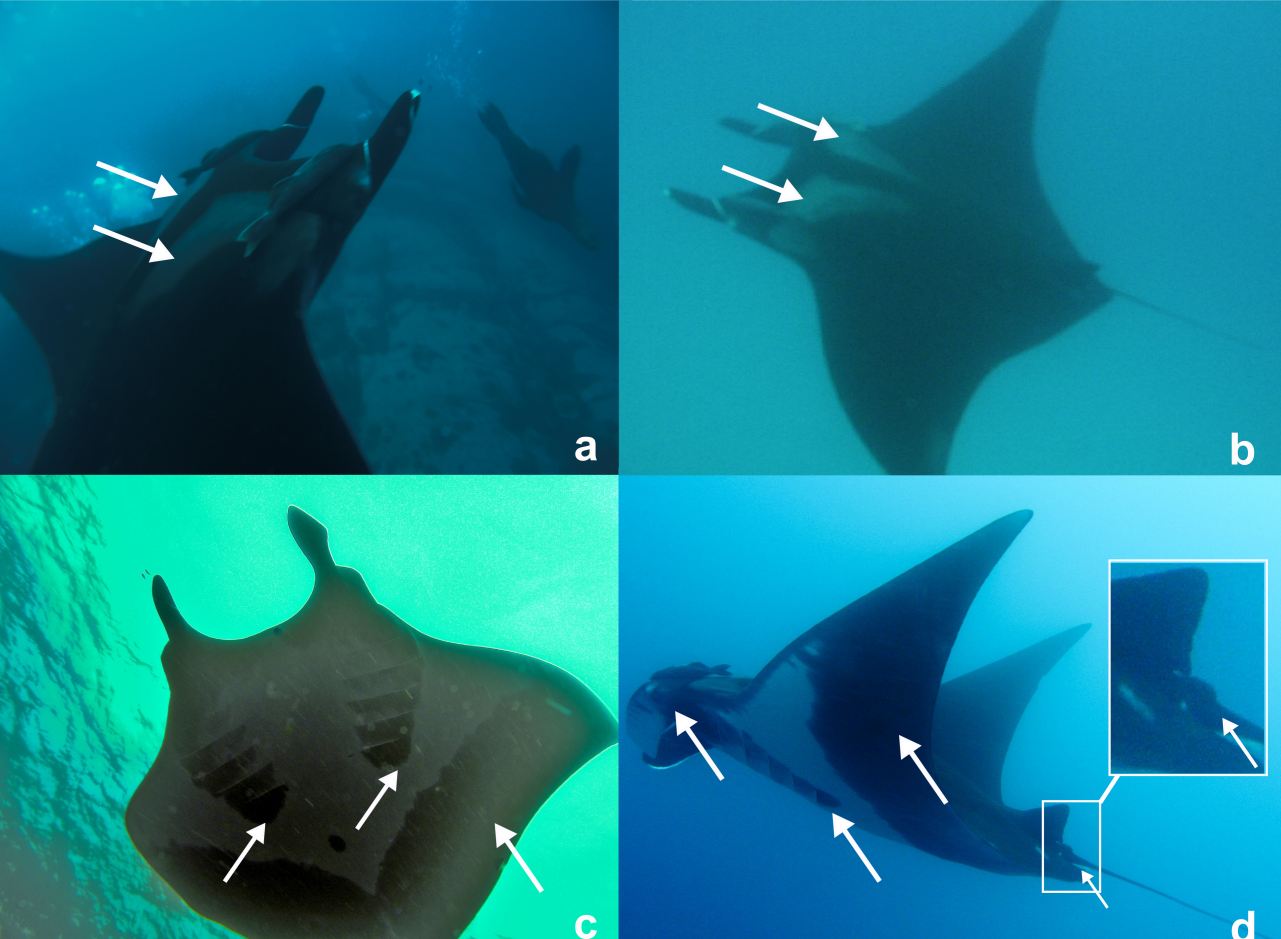
Photographs of a *Manta birostris* (specimen #1) taken off Montague Island on the 5th January 2012 by Peter McGee. White arrows indicate key characters for *M. birostris* as described in Marshall, Compagno & Bennett (2009): (A) and (B) show distinctive dorsal coloration with white shoulder patches with their anterior margins extending medially from spiracles in an approximately straight line parallel to the edge of the mouth; (C) and (D) show large semi-circular spots posterior to the fifth gill slits and grey V-shaped margin along posterior edge of the pectoral fin; and (D) shows dark coloration around mouth and the caudal spine, embedded in a calcified mass and covered with a skin layer, immediately posterior to the dorsal fin (white box).

As part of a larger study, photographs of manta rays were sought from dive clubs, dive instructors, researchers and recreational divers along eastern Australia for photographic identification purposes (see Couturier et al., 2011). Four photographs and two video recordings of a free swimming *M. birostris* were taken by Peter McGee, a recreational diver, off Montague Island (36°15′7.15″S; 150°13′35.19″E; Fig. 1) offshore from Narooma in southern New South Wales (Specimen #1, Fig. 2). The individual was sighted near an Australian fur seal *Arctocephalus pusillus* (Schreber, 1775) colony on the 5th January 2012, swimming at a depth of about 13 m, in 21 °C waters (P McGee, pers. comm., 2013).

**Figure 3:**
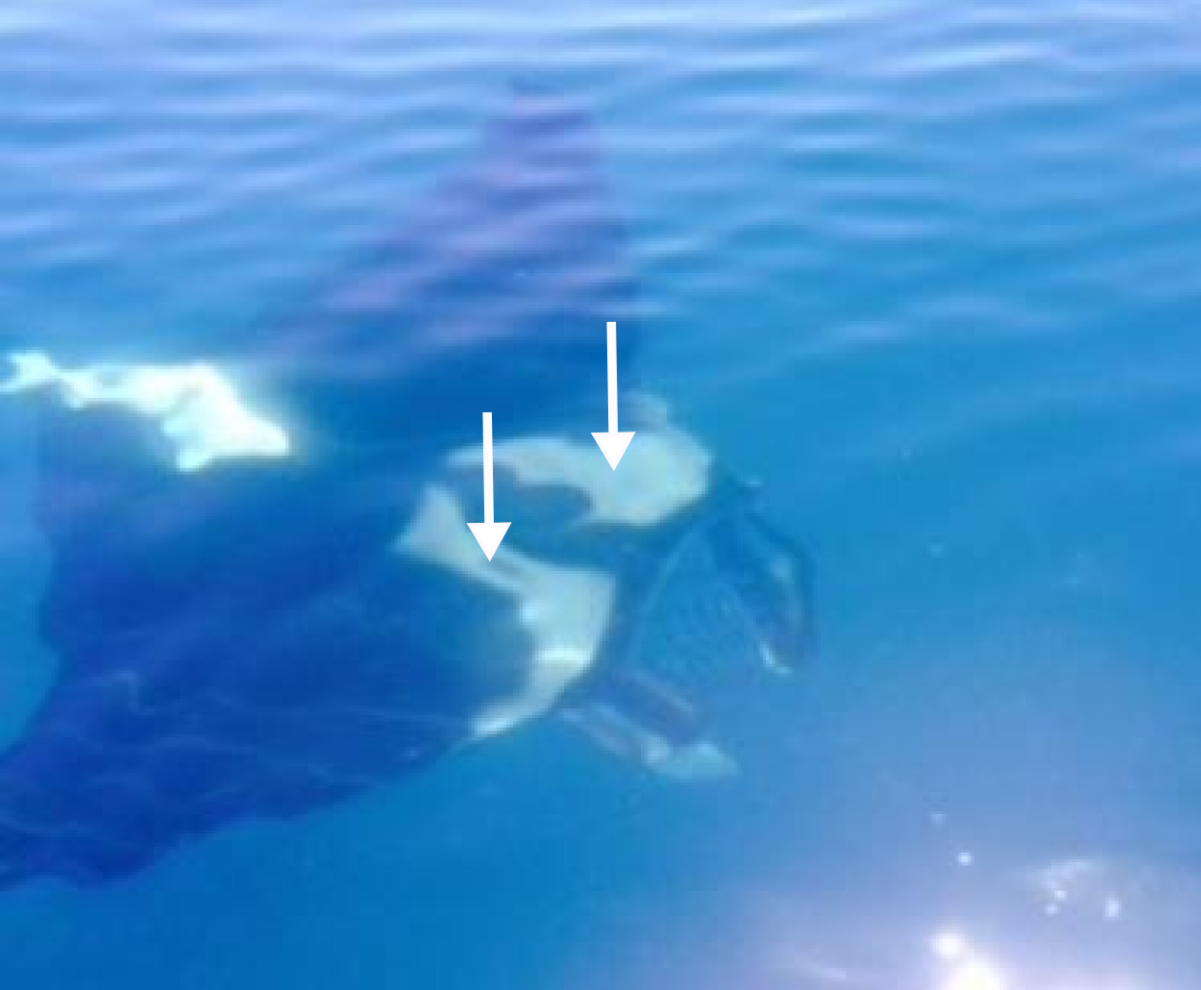
Photographs of a *Manta birostris* (specimen #2) taken off the north east coast of Tasmania on the 26th January 2014 by Leo Miller. White arrows indicate distinctive dorsal white shoulder patches with their anterior margins extending medially from spiracles in an approximately straight line parallel to the edge of the mouth, as key character of the dorsal colouration of *M. birostris* as described in Marshall, Compagno & Bennett (2009).

One photograph of a free swimming *M. birostris* was taken from the surface by Leo Miller, a recreational fisherman, off the north east coast of Tasmania (40°S; 148°E, no precise location given; Fig. 1) on the 26th January 2014. The photograph was submitted to the University of Tasmania Institute of Marine and Antarctic Studies’ Redmap website http://www.redmap.org.au/ (Specimen #2, Fig. 3).

Characters used to confirm identification of *Manta* spp. were terminal mouth, broad head and body coloration. Species identification was based on key morphological features provided by Marshall, Compagno & Bennett (2009), including (1) distinct shoulder patches with triangular shape, (2) presence of a caudal spine, (3) distinctive dark spots on the ventral side over abdominal region, with no spots present medially between the gill slits, (4) prominent semi-circular marking extending posteriorly from both 5th gills and (5) dark-coloured margin on posterior edges of pectoral fins.

## Results and Discussion

Key morphological features, including terminal mouth, broad head, distinctive ventral and dorsal coloration, and presence of caudal spine, could be distinguished from photographs of Specimen #1 (Fig. 2). Together these features allow the specimen to be identified as *M. birostris* and positively differentiated from *M. alfredi*, also known to occur in east Australian waters (Couturier et al., 2011). The distinctive dorsal coloration of Specimen #2 was the only observable morphological feature identifying this individual as *M. birostris* (Fig. 3).

The occurrence of *M. birostris* off Montague Island at ∼36°S in east Australia is consistent with records in south western Pacific Ocean where the species occurs up to 36°S (Duffy & Abbott, 2003; Kashiwagi et al., 2011) and in the south western Atlantic where it occurs up to 34°S (Marshall et al., 2011). Manta ray sightings off Montague Island have been reported in a scuba divers guide (Byron, 1986) and in anecdotal reports (N Coleman & J Van Der Westhuizen, pers. comm., 2012). Manta rays are also commonly advertised as possible diving encounters during austral summer by most dive operators using this dive site (e.g., Narooma Charters, Islands Charters). These unverified sightings were likely to be of *M. birostris* considering that *M. alfredi* distribution range does not appear to extend beyond 30°S worldwide (Marshall, Compagno & Bennett, 2009; Couturier et al., 2014). In addition, *M. alfredi* was not sighted southward of the South Solitary Island (30°12′24.33″S, 153°16′2.52″E) in east Australia despite continuous monitoring effort along the coast over the last 5 years (Couturier et al., 2011; Couturier et al., 2014).

The scarce information available on *M. birostris* migratory ecology suggests that its movements are timed with seasonal oceanographic events known to enhance productivity. Seasonal occurrence of the species off south-eastern Brazil was associated with a low salinity coastal front (Luiz et al., 2009), while movements of tagged manta rays in the Gulf of Mexico were linked to seasonal upwelling events and thermal fronts (Graham et al., 2012). *Manta birostris* and several *Mobula* spp. also occur off North East New Zealand during summer months, which coincide with the path and flow of the East Auckland Current (Duffy & Abbott, 2003).

The occurrence of *M. birostris* off Montague Island may be linked to regional circulation patterns and productive oceanographic events during summer. The East Australian Current (EAC) flows pole-ward along the east Australian coast and its main EAC jet bifurcates abruptly to the east at ∼32°S. About a third of the main EAC jet continues south into the Tasman Sea, towards Montague Island, as a series of dynamic eddies (Ridgway & Godfrey, 1997; Roughan et al., 2011). Enhanced nutrient concentrations and upwelling processes have been documented during austral spring and summer south of the separation point where Montague Island is located (e.g., Oke & Middleton, 2001; Roughan & Middleton, 2004; Ridgway, 2007). These conditions generate ephemeral but highly productive phytoplankton blooms along the coast (Hallegraeff & Jeffrey, 1993; Bax et al., 2001), that likely boost the abundance of zooplankton prey. Humpback whales *Megaptera novaeangliae* regularly feed on small pelagic fish and coastal krill *Nyctiphanes australia* along the southeast Australian coast during their southward migration (Stamation et al., 2007). It is probable that *M. birostris* also occur in this area during warmer months to exploit local productivity events.

The occurrence of *M. birostris* off north east Tasmania at ∼40°S is the newly-extended southern-most record for the species. This sighting may be linked to exceptional oceanographic conditions occurring in the area at the time of the sighting or a response to warming waters by climate-driven changes. South-east Australia is a global warming hotspot where the sea surface temperatures have been increasing up to 3 times the global average rate over the past 50 years, and are projected to rise further into the future (Ridgeway & Hill, 2012; Wu et al., 2012; Hobday & Pecl, 2014; Oliver et al., 2014). Southward range extensions have already been reported in this region for plankton communities, macroalgae, macro-invertebrates and fish (Johnson et al., 2011; Last et al., 2011; Ridgeway & Hill, 2012). Sea surface temperatures around the sighting area usually vary between 12 °C in winter and 17 °C in summer (Condie & Dunn, 2006). In warm years, temperatures were reported to increase up to 2 °C above average temperatures recorded 60 years ago due to circulation changes of the EAC (Ridgway, 2007; Ridgeway & Hill, 2012). Although *M. birostris* may tolerate low temperature for short periods of time (e.g., during deep dives), its distribution in tropical and subtropical waters suggest a preference for temperatures above 17 °C (Marshall et al., 2011). It is possible that at the time of the sighting the EAC flow had extended southward along the Tasmanian coast with increased strength (Ridgeway & Hill, 2012; Oliver & Holbrook, 2014), engendering favourable environmental conditions for *M. birostris*. In addition to providing a suitable thermal habitat, the intrusion of warmer waters along the east Tasmanian coast may trigger productivity events (Matear et al., 2013), providing new food resources for the species.

Based on these observations, we confirm the presence of *M. birostris* for the first time in east Australian waters, increasing the known range of the species. The scarcity of recorded observations of *M. birostris* compared to *M. alfredi*, despite vibrant diving and boating activities along the ∼4,000 km east Australian coastline, suggests that the species is rare in the area. It is also possible that the species occupies and traverses areas that are not exploited by fisheries and/or tourism and thus remain undetected.
